# Quantifying temporal trends of age-standardized rates with odds

**DOI:** 10.1186/s12963-018-0173-5

**Published:** 2018-12-18

**Authors:** Chuen Seng Tan, Nathalie Støer, Yilin Ning, Ying Chen, Marie Reilly

**Affiliations:** 1 Saw Swee Hock School of Public Health, National University of Singapore and National University Health System, Singapore, Singapore; 2 Department of Medical Epidemiology and Biostatistics, Karolinska Institutet, Stockholm, Sweden; 3 Norwegian National Advisory Unit on Women’s Health, Oslo University Hospital, Oslo, Norway; 4 NUS Graduate School for Integrative Sciences and Engineering, National University of Singapore, Singapore, Singapore; 5 Department of Surgery, Yong Loo Lin School of Medicine, National University of Singapore and National University Hospital System, Singapore, Singapore

**Keywords:** Burden of disease, Population surveillance, Incidence, Mortality, Epidemiology, Calendar time trends, Rank order method

## Abstract

**Background:**

To quantify temporal trends in age-standardized rates of disease, the convention is to fit a linear regression model to log-transformed rates because the slope term provides the estimated annual percentage change. However, such log-transformation is not always appropriate.

**Methods:**

We propose an alternative method using the rank-ordered logit (ROL) model that is indifferent to log-transformation. This method quantifies the temporal trend using odds, a quantity commonly used in epidemiology, and the log-odds corresponds to the scaled slope parameter estimate from linear regression. The ROL method can be implemented by using the commands for proportional hazards regression in any standard statistical package. We apply the ROL method to estimate temporal trends in age-standardized cancer rates worldwide using the cancer incidence data from the Cancer Incidence in Five Continents plus (CI5plus) database for the period 1953 to 2007 and compare the estimates to their scaled counterparts obtained from linear regression with and without log-transformation.

**Results:**

We found a strong concordance in the direction and significance of the temporal trends in cancer incidence estimated by all three approaches, and illustrated how the estimate from the ROL model provides a measure that is comparable to a scaled slope parameter estimated from linear regression.

**Conclusions:**

Our method offers an alternative approach for quantifying temporal trends in incidence or mortality rates in a population that is invariant to transformation, and whose estimate of trend agrees with the scaled slope from a linear regression model.

**Electronic supplementary material:**

The online version of this article (10.1186/s12963-018-0173-5) contains supplementary material, which is available to authorized users.

## Background

Monitoring incidence and mortality rates in a population allows stakeholders in health care to track the burden of the disease. Changes in the population rates over time can help to assess the effectiveness of interventions in public health or health care and also inform projections for future health services. Recent years have seen extensive work in analyses of the global burden of disease, with published estimates of global, regional, and national incidence and prevalence rates of several hundred diseases for a majority of countries, both sex-standardized [1] and for specific sex and age groups [2]. Established methods of assessing trends include age-period-cohort models [3, 4] and the estimated annual percentage change [5]. The estimated annual percentage change (EAPC) has been in use for many years by cancer registries to quantify changes in cancer rates over time and to project future rates [3, 5–7]. Conceptually, EAPC represents the average change in the age-standardized rate (ASR) per year. It is usually computed by estimating the slope of a linear regression (LR) model fitted to the log-transformed ASR. Under this framework, for every one-year increase in calendar time, the ASR is assumed to change by a constant factor when expressed as a percentage of the previous year’s rate. However, the LR model can also be used to model the ASR without log-transformation and the slope term will then correspond to the change in ASR for each calendar year [8]. There is no simple relationship between the slopes from these two models and data analysts need to assess whether the increase is linear or exponential when deciding whether the untransformed or log-transformed ASR is the most appropriate.

In epidemiology, the odds ratio is a commonly used measure of association between a binary outcome and an exposure. In this paper, we propose to use odds to quantify time trends in annual ASRs to eliminate the need to consider whether transformation of ASR is necessary when testing for a temporal trend. This approach involves modeling the ranked ASR values across calendar years using the rank-ordered logit (ROL) regression model to obtain the relevant estimates [9]. We illustrate the method by applying it to data from the Cancer Incidence in Five Continents plus (CI5plus) database, and comparing the estimates we obtain to the scaled estimates from the usual LR models, where the scale parameter is estimated from the standard deviation of the error terms.

## Material and methods

The usual approach used to compute EAPC in incidence rates assumes the log-transformed ASR is linearly related to time and a LR model is fitted to the log-transformed ASR with calendar year as the (continuous) independent variable:

1 $$ {y}_i={\beta}_0+{\beta}_1{x}_i+{\varepsilon}_i, $$

where the subscript *i* represents the *i*-th year (*i* = 1, 2, …, *n*) and the error terms, *ε*_*i*_s, are assumed to be independent and normally distributed with mean 0 and variance ${\sigma}_i^2$ [5, 6]. If the error terms have equal variance (i.e., ${\sigma}_i^2={\sigma}^2$), then simple unweighted least squares provides an estimate of the slope term, *β*_1_. As incidence is represented as a count, the assumption of equal variances may not be reasonable, especially for rare diseases, and a weighted least squares may be more appropriate, where the weight for *y*_*i*_ is ${w}_i=\frac{1}{{\sigma_i}^2}$ (see Supplementary materials and methods for details). In practice, when fitting such models to sparse data, there is a need to account for age strata with no events as the log of zero is undefined.

When a LR of the ASR (i.e., no log-transformation) is used to estimate trend [8], the parameter *β*_1_ in Eq. (1) provides an estimate of the annual increment in the incidence rate. On fitting a LR model to log-transformed rates, the EAPC is given by the following transformation of the coefficient (*β*_1_):

2 $$ \mathrm{EAPC}=100\ast \left[\exp \left\{{\beta}_1\right\}-1\right]. $$

### The rank-ordered logit model

The ROL model was originally developed in marketing research for modelling an individual’s preferences for *n* products [9]. The model is linear as in Eq. (1), but the error terms are assumed to be extreme value type 1 (EVT1) distributed with location *μ* = 0 and scale λ = 1 (i.e., standard EVT1 distributed). Under these assumptions, *β*_1_ can be estimated from the ranked observations based on:

3 $$ \mathit{\Pr}\left({y}_1>{y}_2>\dots >{y}_n\right)={\prod}_{i=1}^{n-1}\frac{\mathit{\exp}\left\{{\beta}_1{x}_i\right\}}{\sum_{j=i}^n\mathit{\exp}\left\{{\beta}_1{x}_j\ \right\}}. $$

In marketing research applications, the *β*_1_ parameter indicates the association between a feature of the products and the individual’s preference: for example, if a decrease in the price of a product is associated with an increase in its preference, then exp{*β*_1_} represents the odds of a higher rank (or preference) when the price decreases by one unit. When the error term assumption is fulfilled, the estimate of *β*_1_ also has the usual linear interpretation as in Eq. (1). Note that Eq. (3) is the familiar partial likelihood of a Cox-regression model [10–12]. Hence, the ROL model can be implemented using standard statistical software by using the commands provided for Cox regression analysis.

In applying ROL models to time trend analysis of incidence rates, the ASR (i.e., *y*) is used to rank the calendar years. Thus the calendar year is the explanatory variable (i.e., *x*) and the ROL model provides an estimate of the association between calendar year and the magnitude (or rank) of the ASR. Since the ROL is indifferent to any transformation of the outcome that preserves the ordering, the odds of the subsequent calendar year having a higher value (or rank) than the current year is exp{*β*_1_}, regardless of whether or not the ASR is log transformed.

### The scale parameter, ***λ***, for the slope term, ***β***_**1**_, from the linear regression model

The ROL model specifically assumes standard EVT1 distributed error terms, thus the variance equals *π*^2^/6. In contrast, the variance of the error terms in the LR model is not specified a priori but estimated from the data. Because of this, the *β*_1_ estimates from the two regression models are not comparable. We can overcome this by scaling the outcome in the LR model (and thus scaling *β*_1_).

For a linear model such as that in Eq. (1), if the error terms are independently and identically distributed with an EVT1 distribution with *μ = 0* and λ > 0, the variance of the error terms (and consequently the variance of the outcome) is given by,

4 $$ {\sigma}_y^2={\uplambda}^2{\pi}^2/6. $$

Hence, we can estimate a scale-like parameter, λ, from the error terms obtained from the usual LR (assuming these are independent and normally distributed with mean 0 and *σ* > 0) by equating the variance expression in Eq. (4) with the estimate of *σ* from the LR model and solving for λ, i.e., $\uplambda =\sqrt{6}\sigma /\pi$.

Scaling the outcome variable *y* from Eq. (1) by λ gives ${y}_i^{\ast }={y}_i/\uplambda ={\beta}_0^{\ast }+{\beta}_1^{\ast }{x}_i+{\varepsilon}_i^{\ast }$ where ${\varepsilon}_i^{\ast }={\varepsilon}_i/\uplambda$ mimics the standard EVT1 distribution assumption of the error terms in the ROL in Eq. (3). Hence, the scaled slope parameter ${\beta}_1^{\ast }={\beta}_1/\uplambda$ from Eq. (1) represents the slope parameter in the ROL model in Eq. (3). Thus, the proposed scaled slope from LR has a similar interpretation to the log-odds in Eq. (3). Thus, we have provided a heuristic argument for scaling the slope from a simple (unweighted) LR where the error variance is represented by a single parameter, *σ*. Extending this to weighted LR would require a single value to represent the variation of the error terms. For simplicity, we propose using the mean of the standard deviations in the different calendar years (i.e., $\sigma =\sum \limits_{i=1}^n{\sigma}_i/n$) to represent the overall underlying variation over the time-period of study.

### Application to cancer data

The CI5plus database has annual incidence rates for 27 cancer sites in 118 populations from 1953 to 2007 with calendar periods of coverage varying for different populations. With the exception of cancers of the breast, cervix uteri, corpus uteri, and ovary and other uterine adnexa in females, and cancer of the prostate and testis in males, cancers at all sites are reported separately for males and females. Yearly incident cancer cases, *c*_*ij*_s, and population denominators, *n*_*ij*_s, aggregated by five-year age groups provide incidence rates suitable for performing time trend analysis where i and j denote the *i*-th calendar year and *j*-th age group. We harmonized all incidence rates and denominators using 16 age-groups (0–4, 5–9, …, 70–74, 75+), and used the Segi world standard population, *s*_*j*_s, to compute the ASRs [13]. We replaced any ASR of zero with half the value of the smallest non-zero ASR in the database for the cancer site(s) being analyzed. From the 27 cancer sites (four of them gender-specific) in 118 populations, we had a total of 5900 trends for analysis. In addition to site and sex-specific cancers, we also considered all sites excluding non-melanoma skin cancer.

We applied the three approaches outlined in the previous section to these worldwide cancer rates. The first approach was the LR of the log-transformed rates (LR-ln), the second approach was the LR of the untransformed rates (LR-un), and the third approach was our proposed ROL regression model on the ranked rates. The LR models were fit using weighted least squares. The estimates of trend obtained from the three approaches and the corresponding scaled-estimates for LR-ln and LR-un were compared. We inspected the concordance in sign with respect to *p*-values for the scaled and unscaled estimates. Additionally, we reported the results from analysis of the trends stratified by sex to demonstrate the consistency with published work and to highlight important trends. To corroborate the contrasting trends that have been reported for breast cancer in Singapore and Sweden [14, 15], we conducted a specific analysis that compared the incidence rates to illustrate the ROL model’s indifference to transformation and to demonstrate the comparability of the estimates obtained.

All analyses were performed with the statistical package R, version 3.1.2 [16] and the commands are provided in the Supplementary material, together with the commands for implementation in other widely-used statistical software packages (SAS, Stata, SPSS).

## Results

**Fig. 1:**
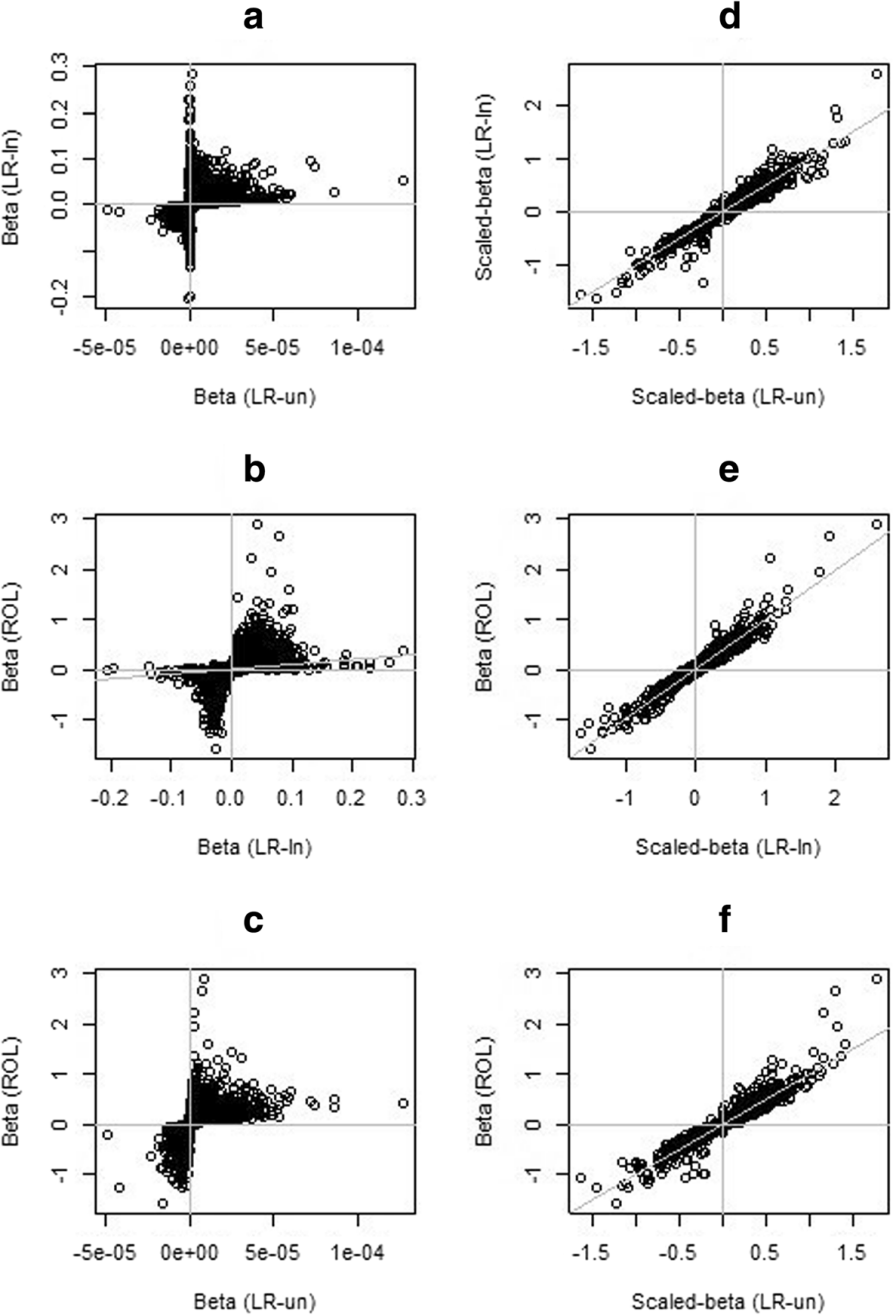
Scatterplots of estimates from weighted linear regression and rank-ordered logit. **a c:** Scatterplots of the slope (β_1_) estimates from weighted linear regression of log-transformed rates (LR-ln) and untransformed rates (LR-un), and β_1_ estimate from rank-ordered logit (ROL). **d-f:** scatterplots of the scaled slope (${\beta}_1^{\ast }$) estimates from LR-ln and LR-un and β_1_ estimate from RO-logit (ROL). The grey horizontal, vertical, and diagonal lines correspond to y = 0, x = 0, and y = x respectively

**Fig. 2:**
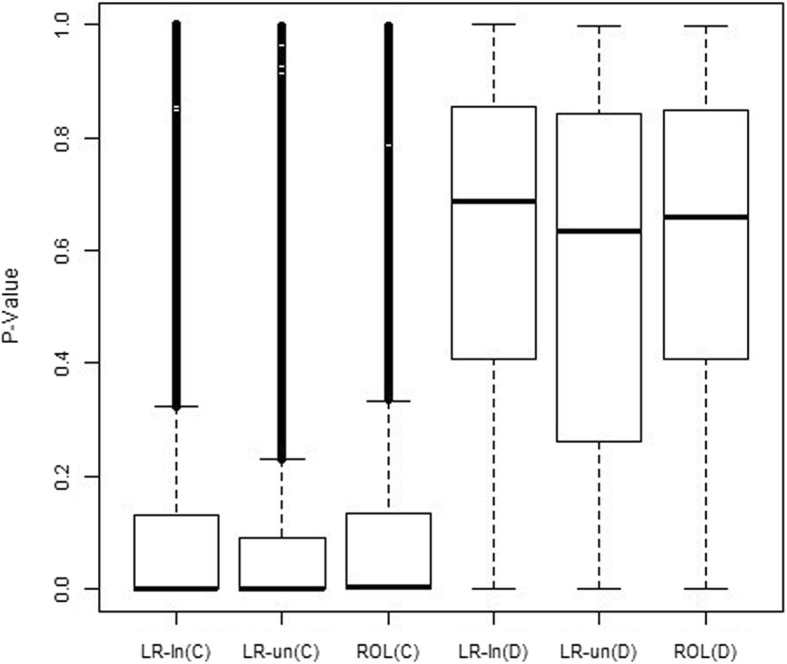
Boxplots of *p*-values from weighted linear regression and rank-ordered logit model. Boxplots show the p-values from testing *β*_1_ = 0 based on concordance (C) and discordance (D) of the signs of the estimated *β*_1_ from all three approaches: weighted linear regression on log-transformed rates (LR-ln) and on untransformed rates (LR-un), and the rank-ordered logit (ROL) model on rates

Figure 1 presents the results from the application of the three approaches to the CI5plus database. The scatterplots in the left column of Fig. 1 provide a pairwise comparison of the estimates of the slope, *β*_1_, from (a) the LR of log-transformed and untransformed ASRs (b) the log-transformed ASRs and the ROL, and (c) the untransformed ASRs and the ROL. As expected, these plots did not exhibit a clear relationship between the estimates, although there was a high concordance in the signs of the estimates across the three approaches, with 5325 out of 5900 combinations (90.3%) having the same sign across all three approaches. With regard to inference concerning the direction of temporal trends, the *p*-values corresponding to these concordant scenarios were lower than those from scenarios where the signs were discordant (see Fig. 2). Examining the scaled-estimates, ${\beta}_1^{\ast }$, from the linear regression of untransformed and log-transformed rates and comparing them to each other (Fig. 1 (d)) and comparing each of these estimates to the *β*_1_ estimate from the ROL analysis (Fig. 1 (e) and (f)), we see that the scatterplots exhibit a pronounced linear relationship along the line-of-identity (i.e., the grey diagonal line corresponding to y = x).

On inspection of the divergent points in Fig. 1 (d), (e), and (f), we found several of these were for prostate cancer where the introduction of screening resulted in the familiar “screening effect” feature in the incidence profile so that it is not reasonable to consider a linear fit. One unexpected disagreement was for thyroid cancer in New York, whose incidence curve had an apparent screening effect in 2000–2005, and we found that indeed thyroid cancer screening had been offered in New York after the events of 9/11 [17, 18]. For disagreements not due to screening, we found that where the estimates from LR models of untransformed and log-transformed rates disagree, the ROL estimate tends to agree well with the most appropriate LR estimate. These and other divergent points from Fig. 1 are presented in detail in Additional file 1: Figures S1 and S2.

The numerical results from the ROL and LR of the log-transformed rates of sex-specific rates are presented in Table 1, where we do not report results for any cancers where 25% or more of the yearly ASRs were less than 3 per 100,000: cancers of the eye, bone, testis, gallbladder, Hodgkin’s lymphoma and multiple myeloma. The remaining cancers were sorted by the concordance in the significance between the two approaches across the 118 populations. For cancers that affect both genders, the average concordance was used. For “All sites but non-melanoma skin,” the overall concordance between LR-ln and ROL was 84.7% among the 118 populations for both males and females, with an increasing trend in at least 75% of the 118 populations as indicated by the interquartile range excluding an odds of 1 in the ROL and excluding an EAPC value of 0 in the LR-ln analyses respectively. Among the nine cancer sites with more than 70% concordance in significant findings among the 118 populations, two of the three sex-specific cancers (prostate and breast) had an increasing trend in the majority of the populations (≥ 75%) while cancer of the cervix had a decreasing trend in the majority of the populations. For the six cancers affecting both sexes, there was evidence in a majority of populations of an increasing trend for both males and females in cancer of the thyroid and kidney, non-Hodgkin’s lymphoma and non-melanoma skin cancer and a decreasing trend in both sexes for stomach cancer. For lung cancer, there was evidence of an increasing trend in females and decreasing trend in males.

**Table 1:** Comparison of odds and estimated annual percentage change (EAPC)

	Male			Female		
Site^a^	Odds Median (1Q; 3Q)	EAPC Median (1Q; 3Q)	Concordance (%)	Odds Median (1Q; 3Q)	EAPC Median (1Q; 3Q)	Concordance (%)
*All sites but non-melanoma skin*	*1.18 (1.1; 1.29)*	*0.83 (0.43; 1.13)*	*85*	*1.21 (1.13; 1.39)*	*0.71 (0.44; 1.17)*	*85*
Prostate	1.43 (1.22; 1.67)	4.38 (3.14; 5.73)	95			
Breast				1.24 (1.15; 1.46)	1.52 (0.96; 2.11)	88
Stomach	0.79 (0.66; 0.87)	−2.42 (− 3.08; − 1.79)	91	0.83 (0.71; 0.90)	− 2.40 (− 3.20; − 1.64)	82
Lung	0.92 (0.77; 1.02)	− 1.22 (− 1.80; 0.11)	76	1.17 (1.09; 1.33)	1.98 (0.98; 3.56)	78
Cervix uteri				0.85 (0.79; 0.94)	−2.46 (− 3.44; − 1.08)	76
Thyroid	1.10 (1.05; 1.18)	2.75 (1.52; 4.27)	69	1.18 (1.11; 1.34)	3.46 (2.11; 5.54)	84
Kidney etc.	1.16 (1.08; 1.25)	1.93 (1.46; 2.70)	81	1.12 (1.07; 1.18)	2.05 (1.22; 2.45)	70
Non-Hodgkin Lymphoma	1.14 (1.08; 1.21)	1.91 (1.31; 2.44)	75	1.17 (1.10; 1.24)	2.06 (1.43; 2.83)	77
Melanoma of skin	1.22 (1.07; 1.45)	3.46 (2.01; 4.77)	75	1.17 (1.06; 1.32)	2.89 (1.25; 4.37)	70
Liver	1.14 (1.07; 1.27)	2.70 (0.97; 3.67)	78	1.07 (1.01; 1.15)	1.75 (−0.04; 3.01)	54
Colon	1.09 (1.01; 1.25)	0.85 (− 0.01; 2.36)	69	1.04 (0.97; 1.18)	0.29 (−0.38; 1.94)	62
Corpus uteri				1.10 (1.01; 1.19)	0.95 (−0.02; 1.84)	62
Oral cavity & pharynx	0.96 (0.88; 1.02)	−0.67 (−1.6; 0.14)	62	1.04 (0.96; 1.09)	0.64 (−0.92; 1.56)	53
Ovary/other uterine adnexa				0.99 (0.94; 1.05)	−0.29 (− 0.74; 0.54)	50
Esophagus	1.00 (0.92; 1.09)	−0.15 (−1.84; 1.33)	59	0.99 (0.92; 1.04)	−0.34 (−2.13; 0.86)	40
Rectum and anus	1.06 (1.00; 1.15)	0.69 (−0.07; 1.51)	55	1.03 (1.00; 1.09)	0.35 (−0.20; 0.9)	42
Larynx	0.92 (0.84; 0.97)	−1.85 (−2.50; −0.71)	61	1.00 (0.95; 1.03)	−0.47 (−2.21; 1.06)	29
Bladder	1.04 (0.99; 1.11)	0.39 (−0.16; 1.44)	47	1.03 (0.99; 1.09)	0.44 (−0.33; 1.50)	40
Pancreas	1.02 (0.97; 1.06)	0.10 (−0.50; 1.02)	44	1.05 (1.01; 1.10)	0.51 (0.04; 1.81)	36
Leukemia	1.03 (1; 1.07)	0.29 (−0.04; 0.88)	31	1.04 (1.00; 1.08)	0.48 (0.03; 0.98)	37
Brain and central nervous system	1.03 (1; 1.07)	0.49 (−0.13; 0.97)	33	1.03 (1.00; 1.06)	0.54 (−0.07; 1.36)	32

^a^Cancer sites were ordered by the percentage concordance in the significance between the odds from rank-ordered logit and EAPC from the weighted linear regression of log-transformed rates, which indicates the strength and persistence of evidence of a temporal trend across the 118 populations in CI5plus. Abbreviations: *1Q* First quartile, *3Q* Third quartile

For cancer sites with lower concordance in significant findings between LR-ln and ROL, the evidence of an increasing or decreasing trend among the 118 populations is weaker. Only liver cancer in men and uterine cancer in women had an increasing trend of reasonable magnitude (median odds 1.14 and 1.10 respectively). For most of the rarer cancers, the odds estimates from the different populations were close to 1 and the EAPC close to 0.

**Fig. 3:**
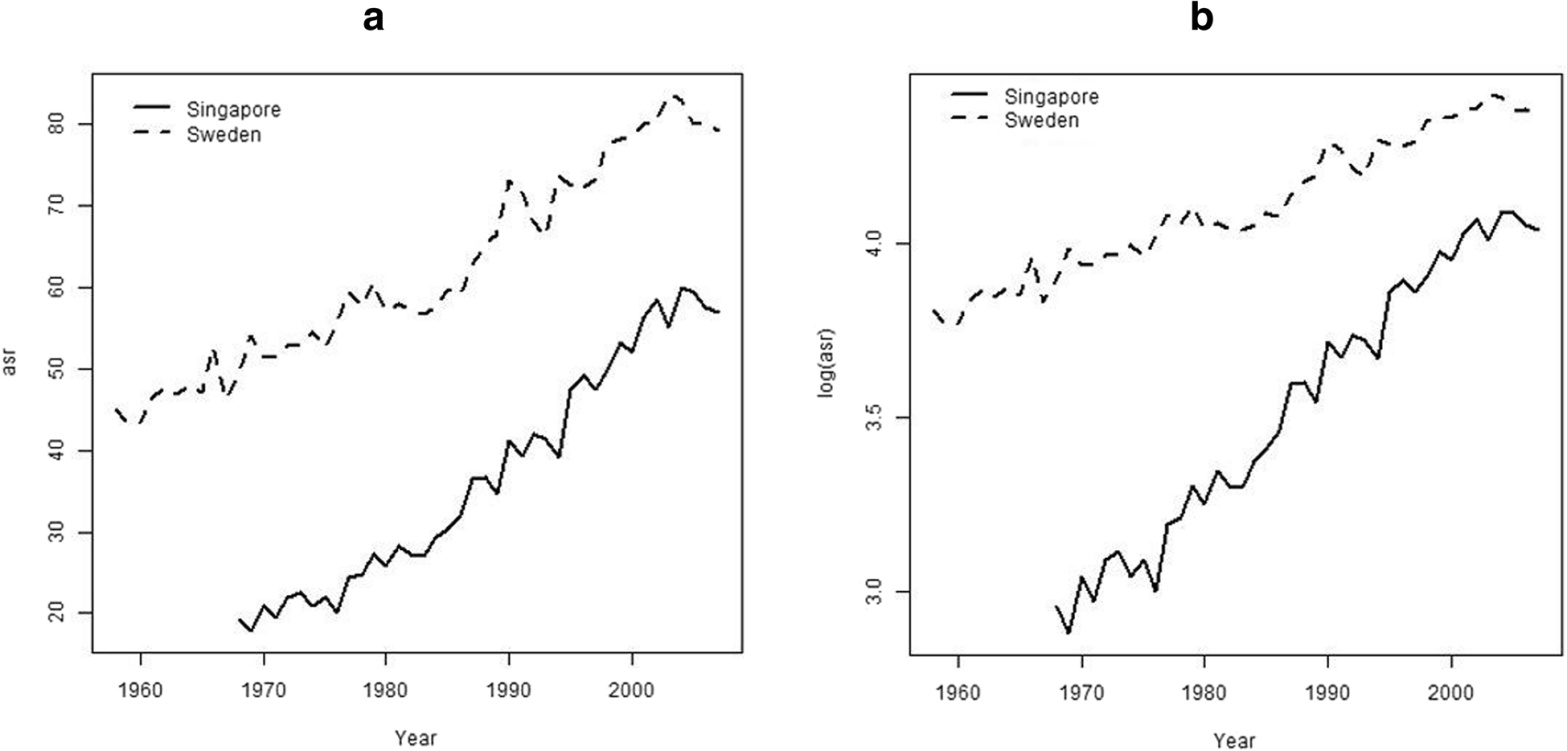
Temporal female breast cancer incidence trends for Singapore (solid line) and Sweden (broken line) using (**a**) untransformed and (**b**) log-transformed age-standardized rates (ASRs)

Figure 3 displays the untransformed and log-transformed ASR of female breast cancer incidence in Singapore and Sweden, suggesting that a linear trend was reasonable for both the untransformed (a) or log-transformed (b) data in both populations. In Table 2, we report the estimates from the LR analysis of both the untransformed and log-transformed rates. The scaled-slope estimates from both analyses were close to the estimates from the ROL analysis in both populations, with slightly better agreement for untransformed rates in the Swedish data. All analyses indicated an increasing trend in breast cancer incidence in both Singapore and Sweden, with a steeper trend in Singapore than in Sweden, consistent with Fig. 3 and with previously published work [14, 15].

**Table 2:** Unscaled and scaled estimates of temporal trend in female breast cancer in Singapore and Sweden

Country	Type	LR-ln	LR-un	ROL
Singapore	Beta	0.03	1.17	
	Scaled-beta	0.51	0.53	0.51
Sweden	Beta	0.01	0.81	
	Scaled-beta	0.42	0.37	0.35

## Discussion

We have described an alternative approach to quantifying temporal trends that is comparable to current practice but with some important advantages. In contrast to much of the published disease trends, which are estimated with specialized models and software [1, 2], our approach uses simple commands available in any standard statistical package and implements a familiar model (Cox proportional hazards regression) to yield an estimate of trend using a measure (the odds) that is familiar in epidemiology. We have provided detailed instructions in the Supplementary material for implementation in several commonly used statistical software packages. The method uses the ROL model, which is commonly used in marketing research but is not a mainstream analytical tool in traditional epidemiology. The usefulness of the model in assessing trends is that it is indifferent to transformations of the age-standardized rates, so there is no need to assess whether the untransformed or log-transformed rates are the most appropriate before proceeding with estimation. This can simplify comparisons across populations where the decision to transform differs.

We applied the method to investigate evidence of temporal trends in site-specific cancer incidence rates in the 118 populations represented in the CI5plus database and compared our results to those from the usual regression models. We found strong concordance in the signs of the estimates and the significance of temporal trends across the three approaches: linear regression (LR) analysis of the untransformed (LR-un) or transformed rates (LR-ln), and ROL. In particular, we found the scaled slopes from the weighted LR analyses to be highly correlated with, and similar to, the *β*_1_ estimates from the ROL model. Unlike the weighted LR whose weights require age-specific population counts and incident cases, our method can be implemented with only annual ASR data. To compare our estimates to those that could be obtained from LR of such data, we conducted a sensitivity analysis using unweighted least squares and obtained very similar results (see Additional file 1: Table S1 and Figure S3) and a high concordance (93.7%: 5526 out of 5900 combinations) in the signs of the estimates across all three approaches (see Additional file 1: Figure S4).

Our analysis demonstrated an increasing trend in many cancers for both men and women, consistent with what has been reported previously [14]. Exceptions, which have also been noted previously, were stomach cancer which had a decreasing trend in both sexes [19], and lung cancer which had an increasing trend in women but decreasing trend in men in a majority of the populations [20]. This lung cancer pattern has been recently observed in many countries and has been attributed to increased smoking among women [21]. The decrease in stomach cancer is harder to explain, but may be due in part to increased exposure to antibiotics [22]. We also found evidence of a decreasing trend in cervical cancer, which has been observed in many populations and been attributed to population-based screening programs [14, 23].

Our comparative analysis of trends can offer additional insights into the health situation within or between specific populations. Our analysis of worldwide cancer incidence rates highlighted a number of interesting features, including the effects of population screening programs (e.g., for prostate cancer), unexpected screening as in New York after the events of 9/11, and the lung cancer profile in Russia (Additional file 1: Figure S2(h)) due to the lack of progress in tobacco control [24].

## Conclusions

The consistency of our estimates from ROL with those from least squares provides empirical evidence that temporal trends in cancer incidence can be represented by odds. The method, which can be seamlessly implemented in standard software, provides a transformation-free alternative that facilitates comparison of trends across different populations in the incidence or mortality rates for any disease or the prevalence rates of known risk factors [25]. For trends that are routinely assessed and reported using regression models, using transformed or untransformed rates, simply including an estimate of the error variance with the reported slope would allow population estimates to be compared with estimates from ROL and all estimates to be combined in meta-analyses, simplifying communication and comparison across populations.

## Additional files

Additional file 1: Supplementary materials. (DOC 5245 kb)

## Abbreviations

ASR: Age-standardized rate
EAPC: Estimated annual percentage change
LR: Linear regression
LR-ln: Linear regression of log-transformed
LR-un: Linear regression of untransformed
ROL: Rank-ordered logit
