# Supplementary material for: Quantifying temporal trends of age-standardized rates with odds
Source: Popul Health Metr. 2018 Dec 18;16:18. doi: 10.1186/s12963-018-0173-5 (PMC6299543; doi:10.1186/s12963-018-0173-5)
Supplement: Supplementary file 1 — Supplementary materials. (DOC 5245 kb) [file 12963_2018_173_MOESM1_ESM.doc]

**Supplementary material and methods**

***Weighted least squares***

For rare diseases, the assumption of equal variance for the age-standardized rates (ASRs) may not be appropriate and a weighted least squares may be preferred, where the rates are weighted by the inverse of their variance. To estimate the variance of ASR, we use the Poisson approximation for each age-specific rate, i.e.,
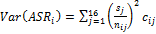
 where *c_ij_* is the observed number of events in age-group *j* in year *i, n_ij_* is the population size in age-group *j* in year *i* and
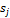
 denotes the appropriate standard population of the *j-th* age group (e.g., the Segi world standard population). For log-transformed rates, these weights can be obtained from a Taylor approximation to estimate the variance of the log-transformed ASR from ASR, i.e.,
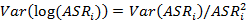
. The inverse of this variance provides the weight for the log-transformed ASR in the *i-th* calendar year.

**Supplementary results**

***Investigation of disparate estimates from the different methods***

The following plots (i.e., Figure S1 and S2) present the results from an in-depth analysis of those cancers where there was disagreement between the estimates from the different methods.

***
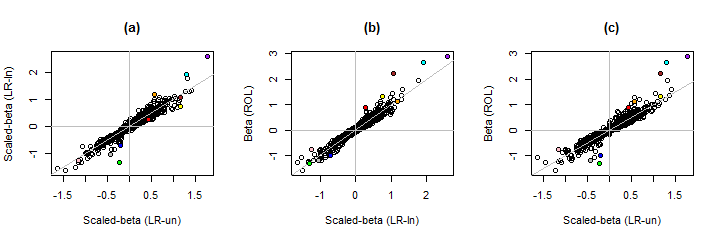
***

| **Colour** | **Registry** | **ICD10** | **Cancer** | **Gender** | **Scaled beta** | | | **R^2^** | |
| --- | --- | --- | --- | --- | --- | --- | --- | --- | --- |
|  |  |  |  |  | **LR-ln** | **LR-un** | **ROL** | **LR-ln** | **LR-un** |
| Red | Denmark | C33-34 | Lung | Female | 0.262 | 0.429 | 0.882 | 0.921 | 0.971 |
| Green | Finland | C16 | Stomach | Male | -1.308 | -0.238 | -1.32 | 0.996 | 0.914 |
| Blue | Finland | C16 | Stomach | Female | -0.695 | -0.204 | -1.004 | 0.987 | 0.879 |
| Yellow | Italy, Romagna | C61 | Prostate | Male | 0.757 | 1.172 | 1.325 | 0.927 | 0.967 |
| Orange | Latvia | C61 | Prostate | Male | 1.168 | 0.571 | 1.114 | 0.97 | 0.876 |
| Brown | The Netherlands | C15 | Esophagus | Male | 1.076 | 1.159 | 2.243 | 0.96 | 0.965 |
| Purple | The Netherlands | C33-34 | Lung | Female | 2.579 | 1.784 | 2.89 | 0.993 | 0.985 |
| Pink | Russia, St Petersburg | C33-34 | Lung | Male | -1.233 | -1.163 | -0.75 | 0.945 | 0.939 |
| Gray | Sweden | C16 | Stomach | Male | -1.042 | -0.439 | -1.211 | 0.993 | 0.964 |
| Cyan | USA, New York State | C73 | Thyroid | Female | 1.906 | 1.315 | 2.639 | 0.98 | 0.958 |

**Figure S1:** The list of scenarios corresponding to the top 5 largest absolute discrepancies in scaled beta between: (a) weighted linear regression of log-transformed rates (LR-ln) and untransformed rates (LR-un), or (b) LR-ln and Rank-Ordered logit (ROL), or (c) LR-un and ROL, with the details of each scenario provided.


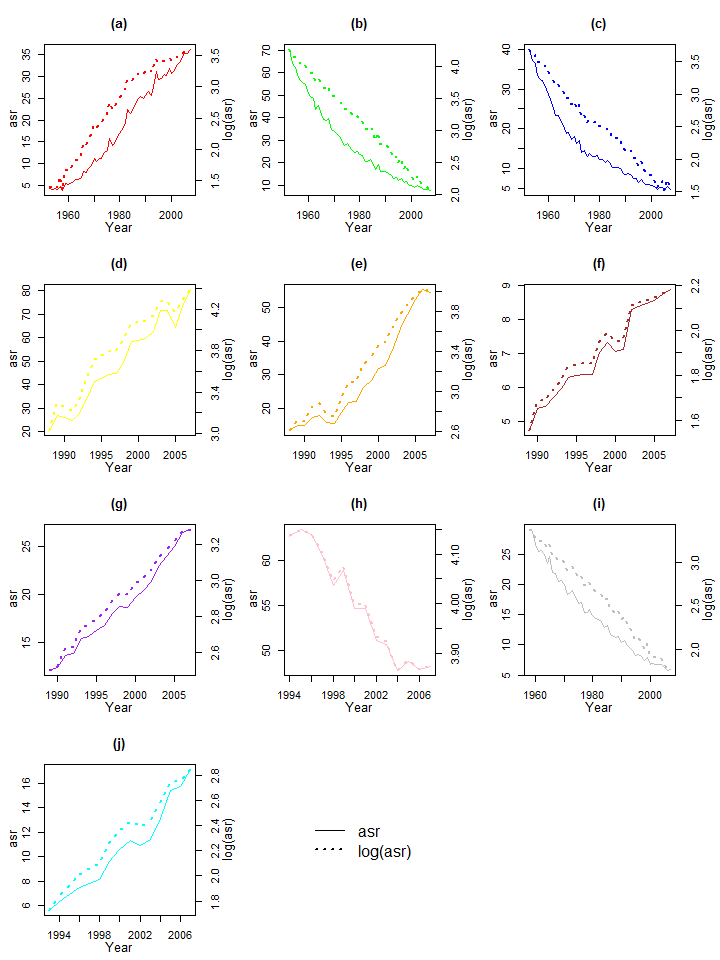


**Figure S2:** The temporal cancer incidence trends for the 10 scenarios listed in Figure S1 for age-standardized rate (asr; solid line) and log-transformed age-standardized rate (log(asr): dotted line). (a) female lung cancer (Denmark), (b) male stomach cancer (Finland), (c) female stomach cancer (Finland), (d) prostate cancer (Italy, Romagna), (e) prostate cancer (Latvia), (f) male esophagus cancer (Netherlands), (g) female lung cancer (Netherlands), (h) male lung cancer (Russia, St Petersburg), (i) male stomach cancer (Sweden), (j) female thyroid cancer (USA, New York State).

***Sensitivity analysis with unweighted linear regression***

The following table (i.e., Table S1) and plots (i.e., Figure S3 and S4) present the results of the sensitivity analysis with unweighted linear regression.

|  | **Male (N=118)** | | | **Female (N=118)** | | |
| --- | --- | --- | --- | --- | --- | --- |
| Site^a^ | Odds  Median (1Q; 3Q) | EAPC  Median (1Q; 3Q ) | Concordance (%) | Odds  Median (1Q; 3Q ) | EAPC  Median (1Q; 3Q ) | Concordance (%) |
| All sites but non-melanoma skin | 1.18 (1.1; 1.29) | 0.86 (0.48; 1.17) | 88 | 1.21 (1.13; 1.39) | 0.77 (0.49; 1.21) | 86 |
|  |  |  |  |  |  |  |
| Prostate | 1.43 (1.22; 1.67) | 4.61 (3.3; 5.8) | 95 |  |  |  |
| Breast |  |  |  | 1.24 (1.15; 1.46) | 1.58 (1.02; 2.21) | 89 |
| Stomach | 0.79 (0.66; -0.87) | -2.45 (-3.15; -1.8) | 91 | 0.83 (0.71; 0.9) | -2.38 (-3.21; -1.66) | 81 |
| Lung | 0.92 (0.77; 1.02) | -1.15 (-1.78; 0.15) | 79 | 1.17 (1.09; 1.33) | 2.29 (1.11; 4) | 80 |
| Kidney etc. | 1.16 (1.08; 1.25) | 1.97 (1.5; 3.11) | 82 | 1.12 (1.07; 1.18) | 2.05 (1.44; 3.06) | 75 |
| Cervix uteri |  |  |  | 0.85 (0.79; 0.94) | -2.4 (-3.46; -1.07) | 79 |
| Non-Hodgkin lymphoma | 1.14 (1.08; 1.21) | 2.23 (1.51; 2.89) | 78 | 1.17 (1.1; 1.24) | 2.31 (1.68; 3.31) | 79 |
| Thyroid | 1.1 (1.05; 1.18) | 2.89 (1.9; 5.45) | 70 | 1.18 (1.11; 1.34) | 3.31 (2.33; 5.88) | 85 |
| Melanoma of skin | 1.22 (1.07; 1.45) | 3.9 (2.7; 5.36) | 76 | 1.17 (1.06; 1.32) | 3.41 (1.79; 4.7) | 74 |
| Liver | 1.14 (1.07; 1.27) | 2.95 (1.38; 3.85) | 81 | 1.07 (1.01; 1.15) | 2.3 (0.32; 3.58) | 58 |
| Colon | 1.09 (1.01; 1.25) | 1.01 (0.11; 2.63) | 71 | 1.04 (0.97; 1.18) | 0.38 (-0.36; 2.17) | 64 |
| Corpus uteri |  |  |  | 1.1 (1.01; 1.19) | 0.99 (-0.04; 2.01) | 63 |
| Oral cavity and pharynx | 0.96 (0.88; 1.02) | -0.66 (-1.46; 0.37) | 62 | 1.04 (0.96; 1.09) | 0.72 (-0.89; 1.65) | 53 |
| Ovary and other uterine adnexa |  |  |  | 0.99 (0.94; 1.05) | -0.22 (-0.73; 0.61) | 54 |
| Rectum and anus | 1.06 (1; 1.15) | 0.76 (-0.03; 1.75) | 60 | 1.03 (1; 1.09) | 0.41 (-0.13; 1.11) | 46 |
| Esophagus | 1 (0.92; 1.09) | 0.03 (-1.76; 1.45) | 60 | 0.99 (0.92; 1.04) | -0.23 (-2.02; 1.52) | 42 |
| Bladder | 1.04 (0.99; 1.11) | 0.46 (-0.15; 1.6) | 51 | 1.03 (0.99; 1.09) | 0.53 (-0.23; 1.76) | 43 |
| Larynx | 0.92 (0.84; 0.97) | -1.77 (-2.57; -0.64) | 61 | 1 (0.95; 1.03) | -0.34 (-2.56; 1.9) | 31 |
| Pancreas | 1.02 (0.97; 1.06) | 0.24 (-0.51; 1.04) | 47 | 1.05 (1.01; 1.1) | 0.53 (0.06; 2.22) | 43 |
| Leukemia | 1.03 (1; 1.07) | 0.39 (0.04; 0.98) | 34 | 1.04 (1; 1.08) | 0.56 (0.1; 1.15) | 41 |
| Brain and central nervous system | 1.03 (1; 1.07) | 0.57 (-0.08; 1.31) | 36 | 1.03 (1; 1.06) | 0.74 (0.02; 1.76) | 36 |

**Table S1: Sensitivity analysis:** comparison of odds and estimated annual percentage change (EAPC**)** from an unweighted regression analysis**.** ^a^ Cancer sites were ordered by the percentage of concordance in the significance between the odds from rank-ordered logit and EAPC from the unweighted linear regression of log-transformed rates which indicates the strength and persistence of evidence of a temporal trend across the 118 populations in CI5plus. Abbreviations: 1Q: First quartile, 3Q: Third quartile.

**
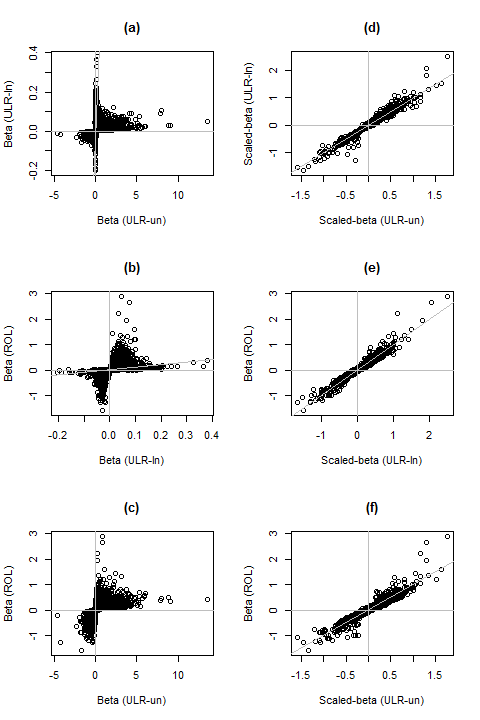
**

**Figure S3:** Scatterplots of estimates from unweighted linear regression and rank-ordered logit. (a) – (c): Scatterplots of the slope
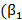
) estimates from unweighted linear regression of log-transformed rates (ULR-ln) and untransformed rates (ULR-un), and
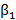
 estimate from rank-ordered logit (ROL). (d) – (f): scatterplots of the scaled slope
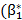
) estimates from ULR-ln and ULR-un and
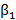
 estimate from RO-logit (ROL). The grey horizontal, vertical and diagonal lines correspond to y=0, x=0 and y=x respectively.


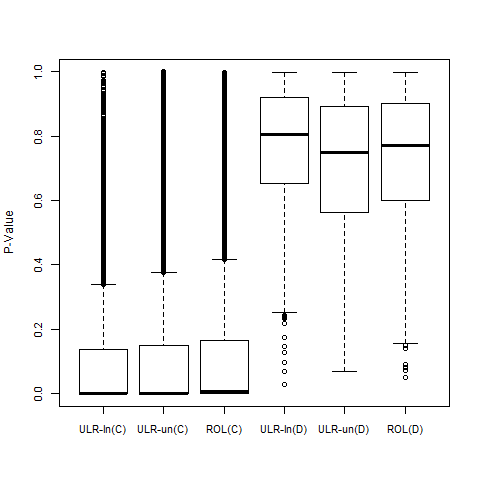


**Figure S4:** Boxplots of p-values from unweighted linear regression and rank-ordered logit. Boxplot of the p-values from testing
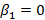
 based on concordance (C) and discordance (D) of the signs of the estimated
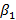
 from all three approaches: unweighted linear regression on log-transformed rates (ULR-ln) and on untransformed rates (ULR-un), and the rank-ordered logit (ROL) model on rates.

**Supplementary instructions**

***Implementing rank-ordered logit analysis of trends in standard statistical software***

Using the age-standardized rates (ASR) of colorectal cancer among Singaporean males as a data example, we illustrate the use of the rank-ordered logit (RO-logit) model to determine the presence of a trend over calendar time. The following table shows an excerpt of the data, which is provided in the file “Colorectal_Singapore_Male_Chinese.csv” at http://blog.nus.edu.sg/dasa/asr_cancer.

| Row number | Year | ASR |
| --- | --- | --- |
| 1 | 1968 | 19.1 |
| 2 | 1969 | 20.9 |
| 3 | 1970 | 22.6 |
| 4 | 1971 | 23.5 |
| 5 | 1972 | 23.5 |
| … | … | … |
| 31 | 1998 | 44.6 |
| 32 | 1999 | 46.2 |
| 33 | 2000 | 42.6 |
| 34 | 2001 | 47.4 |
| 35 | 2002 | 49.2 |

The equivalence of the RO-logit and Cox proportional hazard (Cox-PH) models enables the estimation of the RO-logit parameters using the familiar tools for survival analysis, with the continuous outcome recoded in the reverse order and all indicated as uncensored. For the years with identical values of ASR (i.e., ties) due to rounding, e.g., years 1971 and 1972, we use the Efron or Breslow method.

# ***Implementation in R***

The RO-logit model is implemented by the package ROlogit.

**Step 1:** Install and load the ROlogit package.

| install.packages("ROlogit")  library(ROlogit)  Step 1: Import data.  dat <- read.csv("Colorectal_Singapore_Male_Chinese.csv") |
| --- |

**Step 2:** Implement the RO-logit model using the rologit function from the package. There is no need to recode the outcome because this step is implemented internally in the function. Ties are handled by the Efron method by default.

| model <- rologit(yvar = "ASR", evar = "Year", svar = NULL, dat = dat)  summary(model) |
| --- |

The output from the RO-logit model is the following:

| variable coef scaled.coef exp(coef) se(coef) se(scaled.coef) z Pr(>|z|)  1 Year 0.297 0.787 1.346 0.049 0.131 6.011 0 |
| --- |

The estimated coefficient from the RO-logit model, i.e., the entry under “coef”, is 0.297 (95%CI: 0.201, 0.393; p-value<0.001) which suggests a significant positive trend in ASR where the lower and upper 95%CI is 0.297-1.96×0.049 and 0.297+1.96×0.049 respectively.

# ***Implementation in Stata (using the menus)***

**Step 1:** Import data.

- Go to ***File > Import > Text data (delimited, *.csv, ...)***.
- Under ***File to import*, click *Browse....***
- Select the csv file, **click Open.**

The dialogue box on the right side will show up:

- **Click *OK*.**


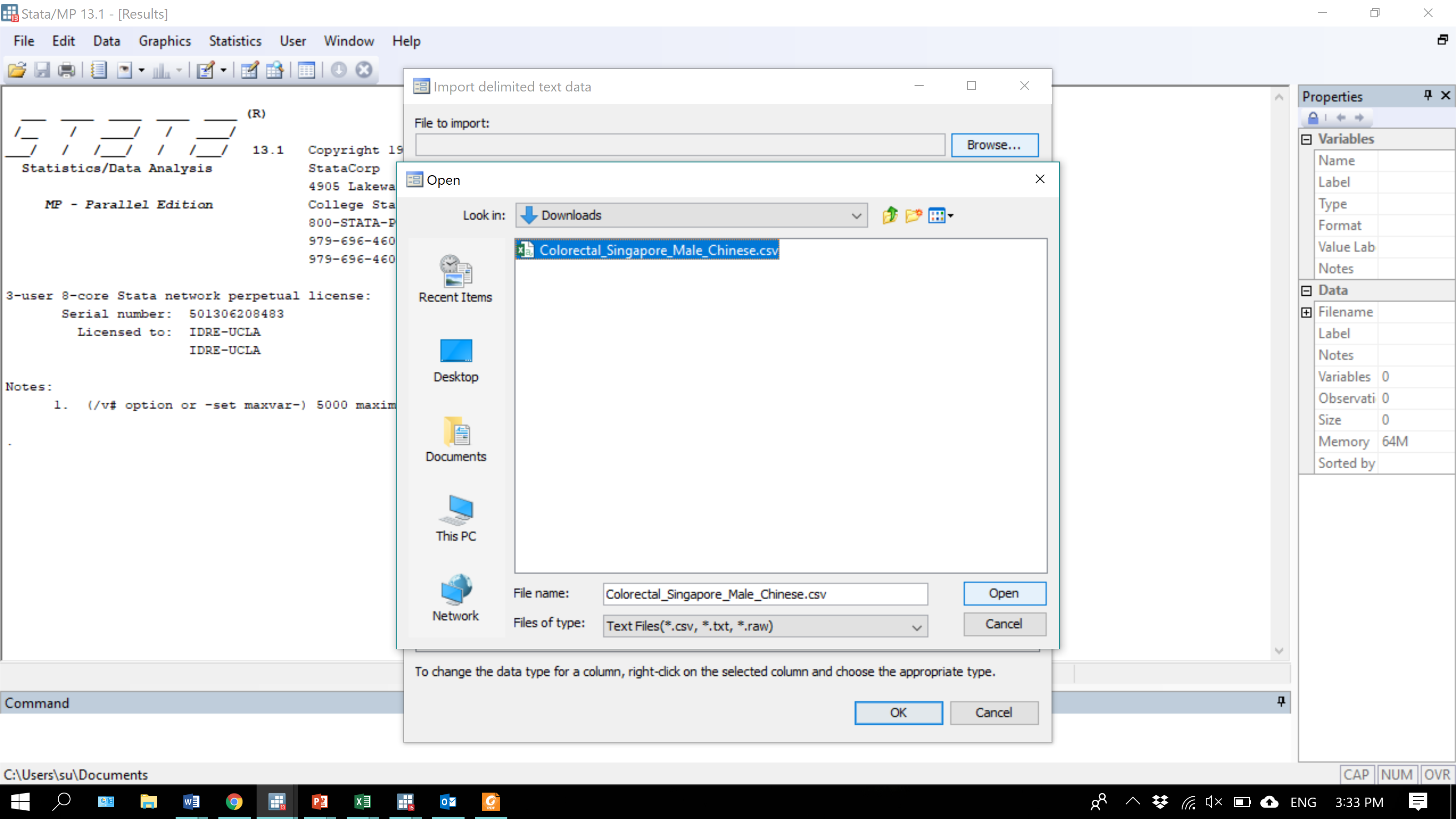

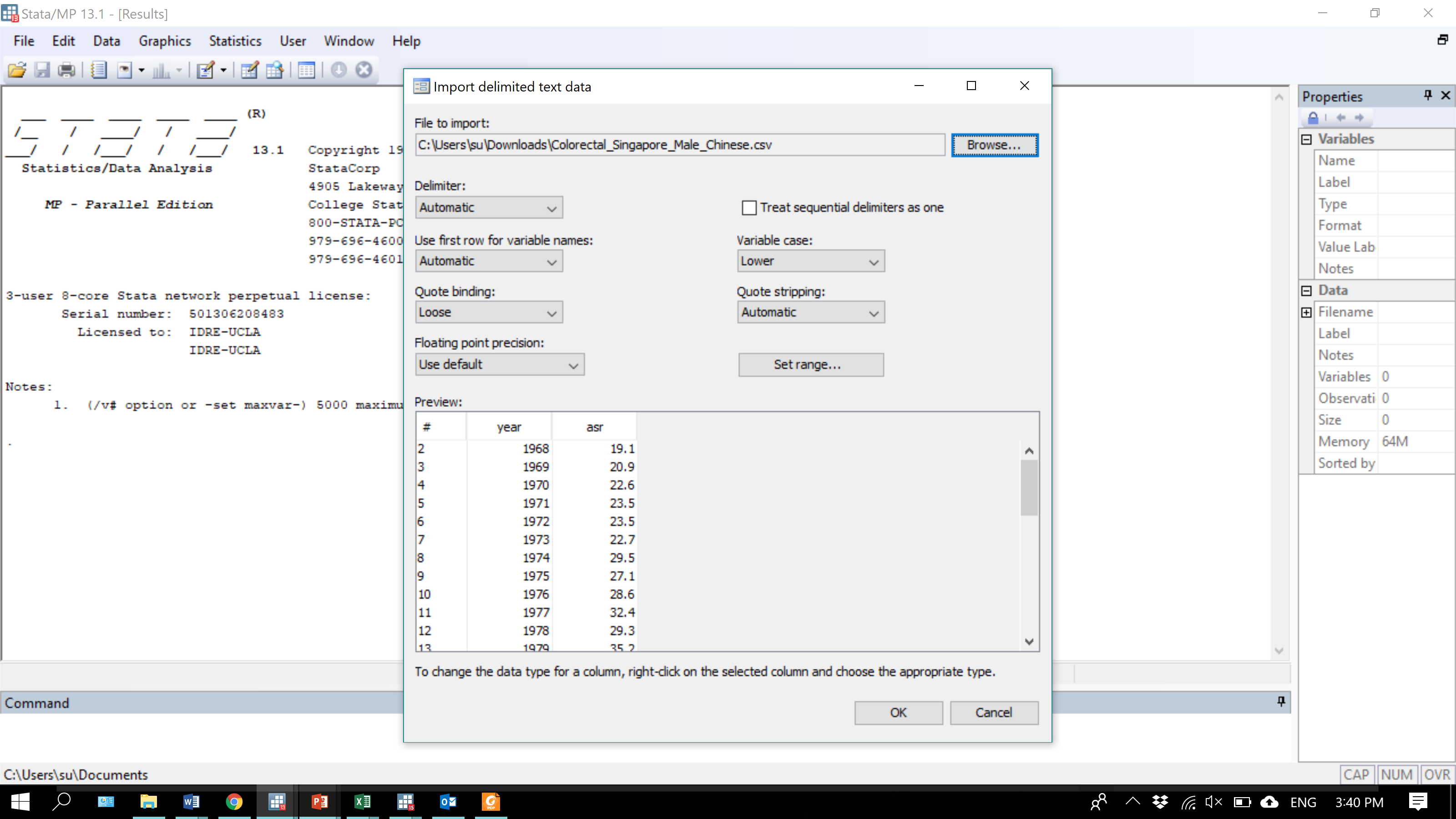


**Step 2:** Recode the outcome, ASR, in the reverse order.

- Go to ***Data > Create or change data > Create new variable (extended)***.
- Under ***Generate variable*, type y.**
- Under ***Egen function,* select *Rank.***
- Under ***Expression*, type -*asr*.**
- **Click *OK*.**


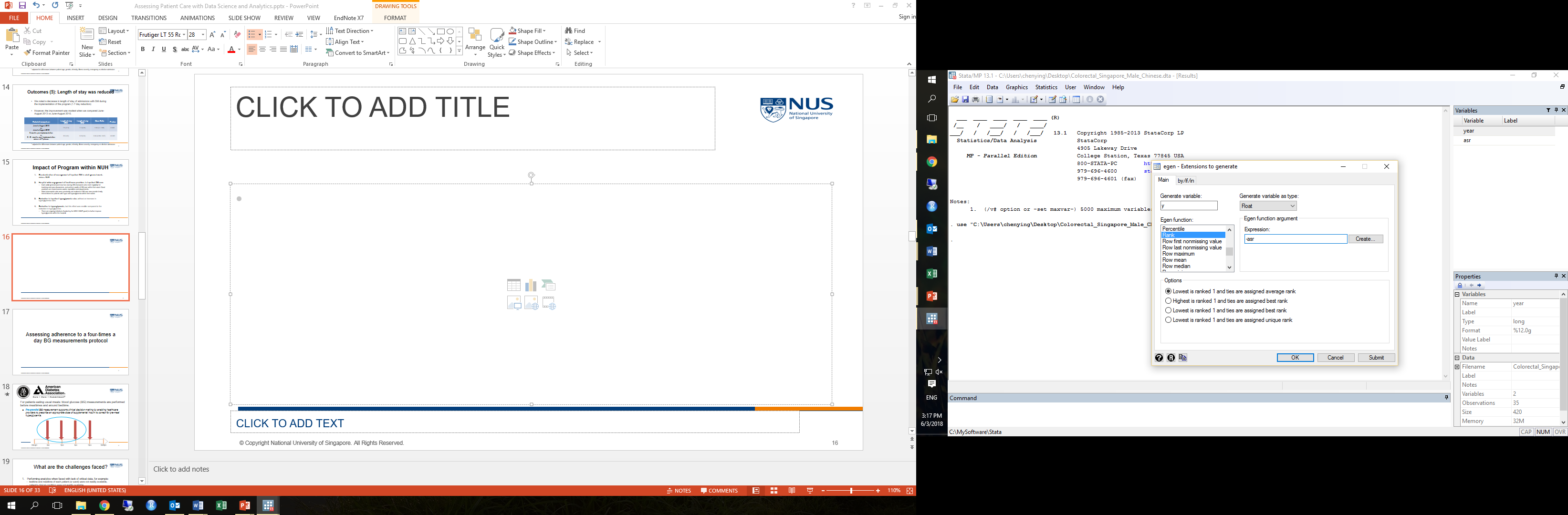


**Step 3:** Implement the RO-logit model by fitting a Cox-PH model to the new outcome, y.

To declare the dataset as survival-time data:

- Go to ***Statistics > Survival analysis > Setup and utilities > Declare data to be survival-time data.***
- Under ***Time variable,* type *y*.**
- **Click *OK*.**


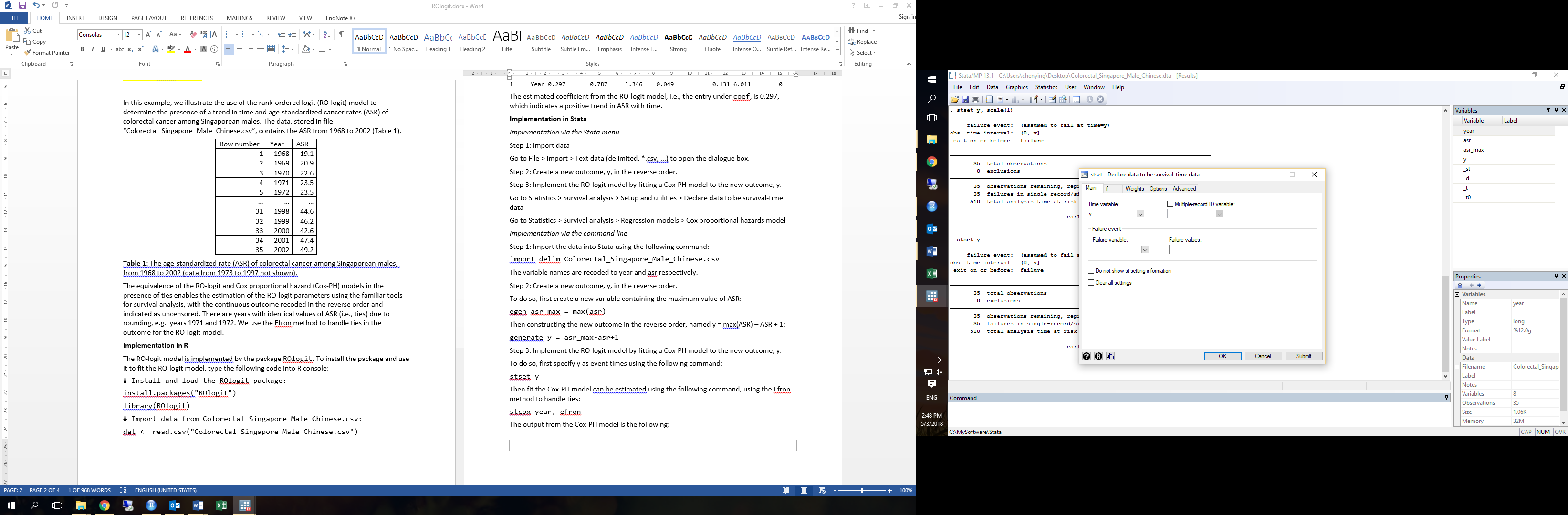


To perform the RO-logit regression, with ties handled by the Efron method:

- Go to ***Statistics > Survival analysis > Regression models > Cox proportional hazards model.***
- Under ***Independent variable,* type *year*.**
- Under ***Method to handle tied failures*, select *Efron*.**
- **Click *OK*.**


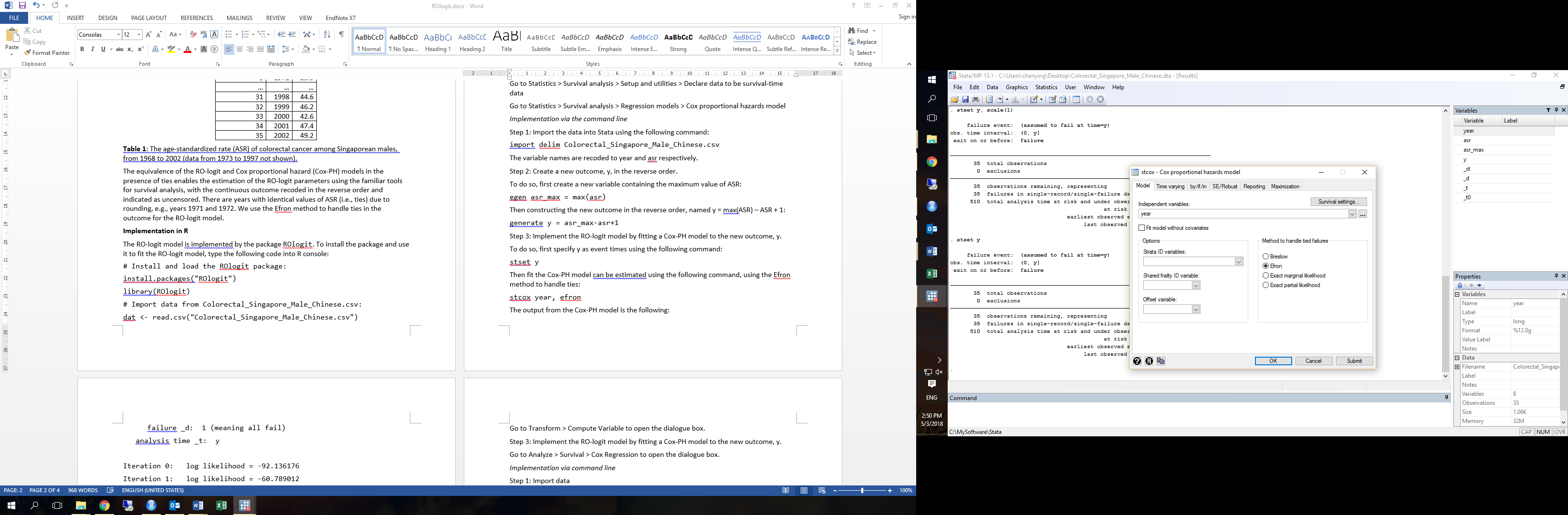


- Under ***Reporting*, check *Report coefficients, not hazard ratios*.**
- **Click *OK***.


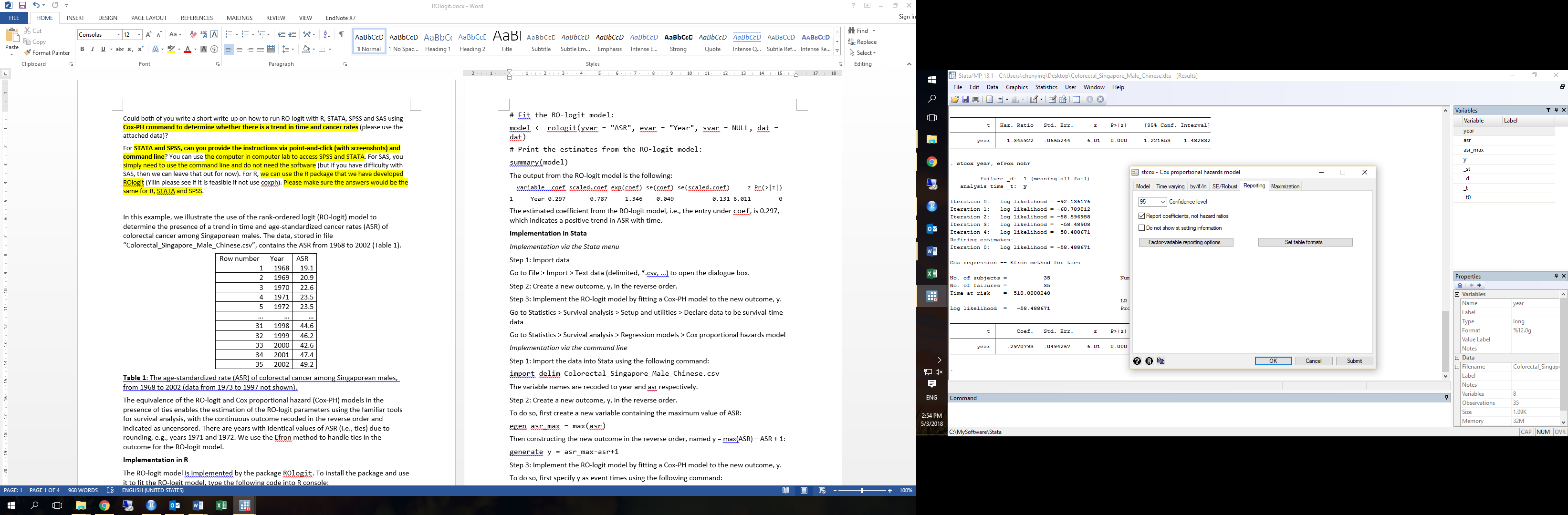


If we do not need to specify a “Failure variable”, Stata will by default assume all entries had failure events. The output is as follows:


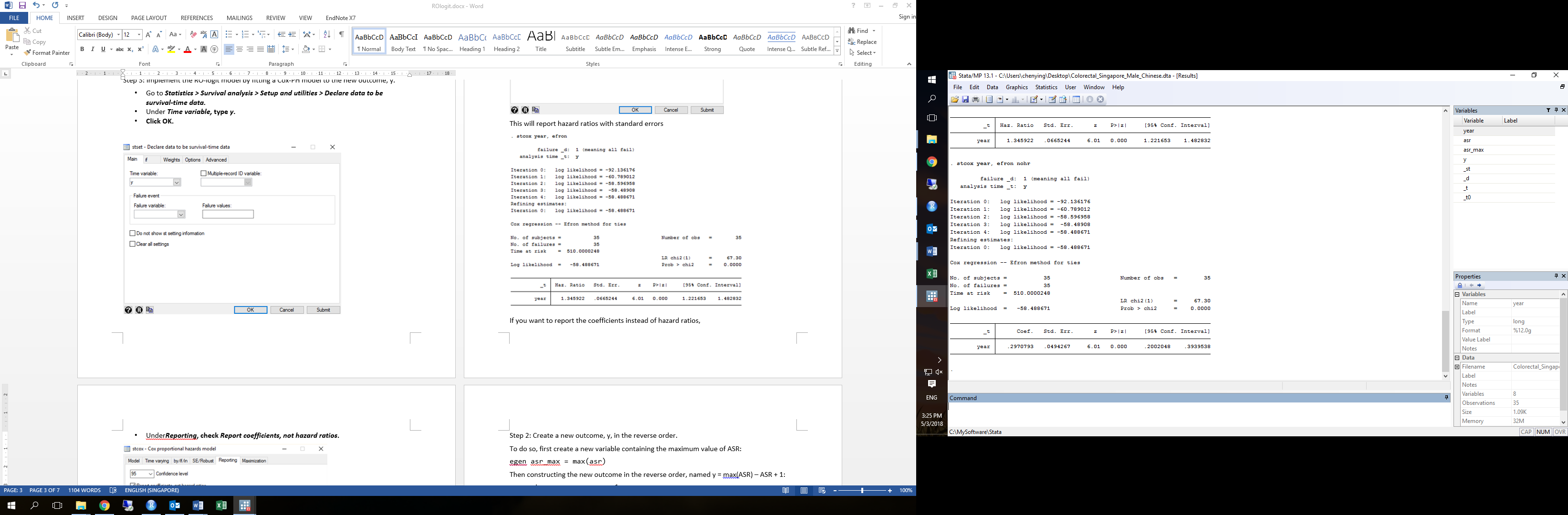


The estimated coefficient from the RO-logit model is 0.297 (95%CI: 0.200, 0.394; p-value<0.001) which suggests a significant positive trend in ASR.

# ***Implementation in Stata via command line***

**Step 1:** Import data.

| import delim Colorectal_Singapore_Male_Chinese.csv |
| --- |

The variables are renamed as year and asr respectively.

**Step 2:** Recode the outcome, ASR, in the reverse order.

| egen y = rank(-asr) |
| --- |

**Step 3:** Implement the RO-logit model by fitting a Cox-PH model to the new outcome, y.

To do so, first specify y as event times using the following command:

| stset y |
| --- |

By default, all the observations are considered as events.

Then fit the Cox-PH model can be estimated using the following command, using the Efron method to handle ties:

| stcox year, efron nohr |
| --- |

# ***Implementation in SPSS using the menus***

**Step 1:** Import data.

- Go to ***File > Import Data > CSV Data***
- Select the csv file, **click *Open*.**

The dialogue box on the right side will show up:

- **Click *OK*.**


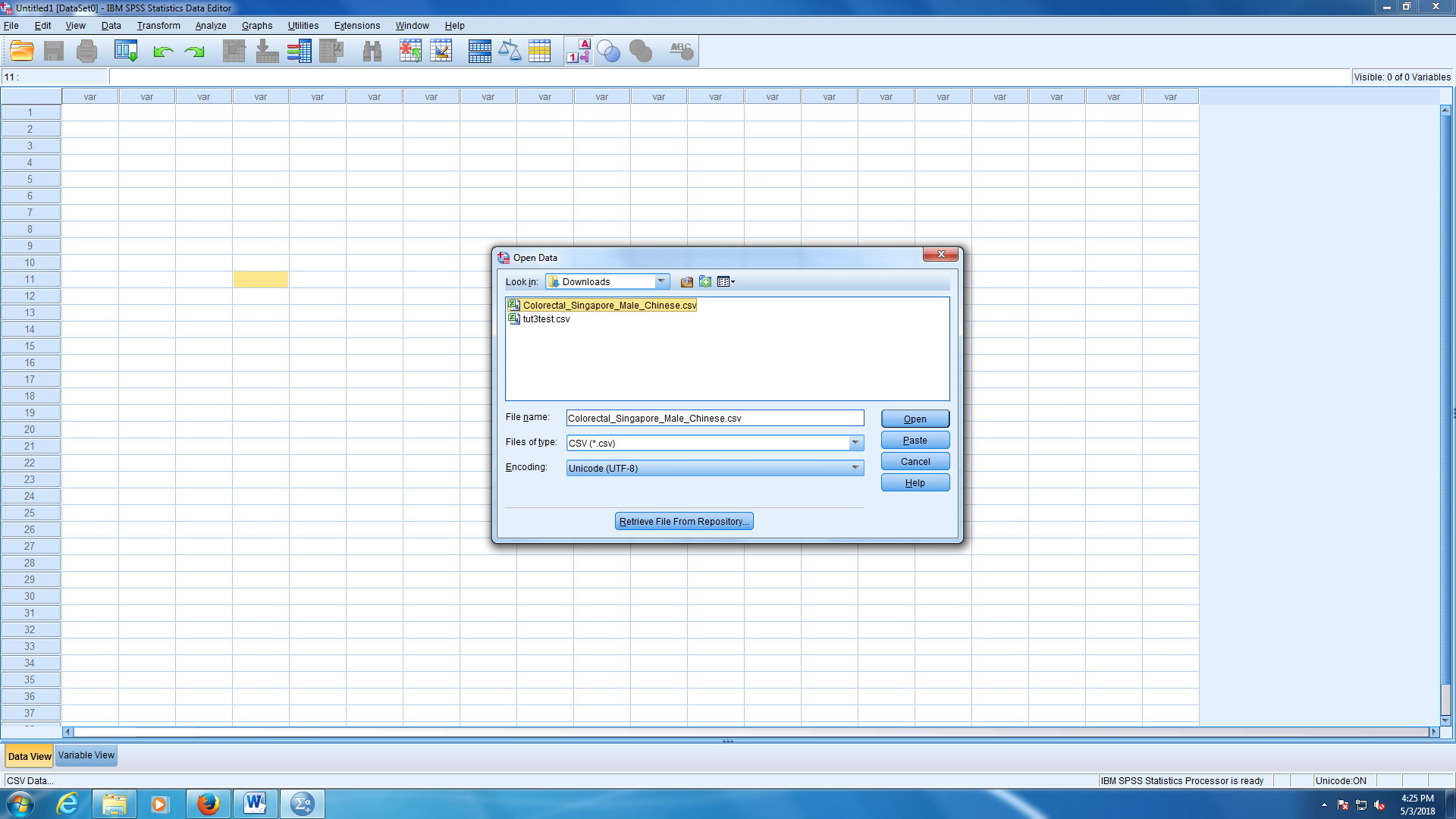

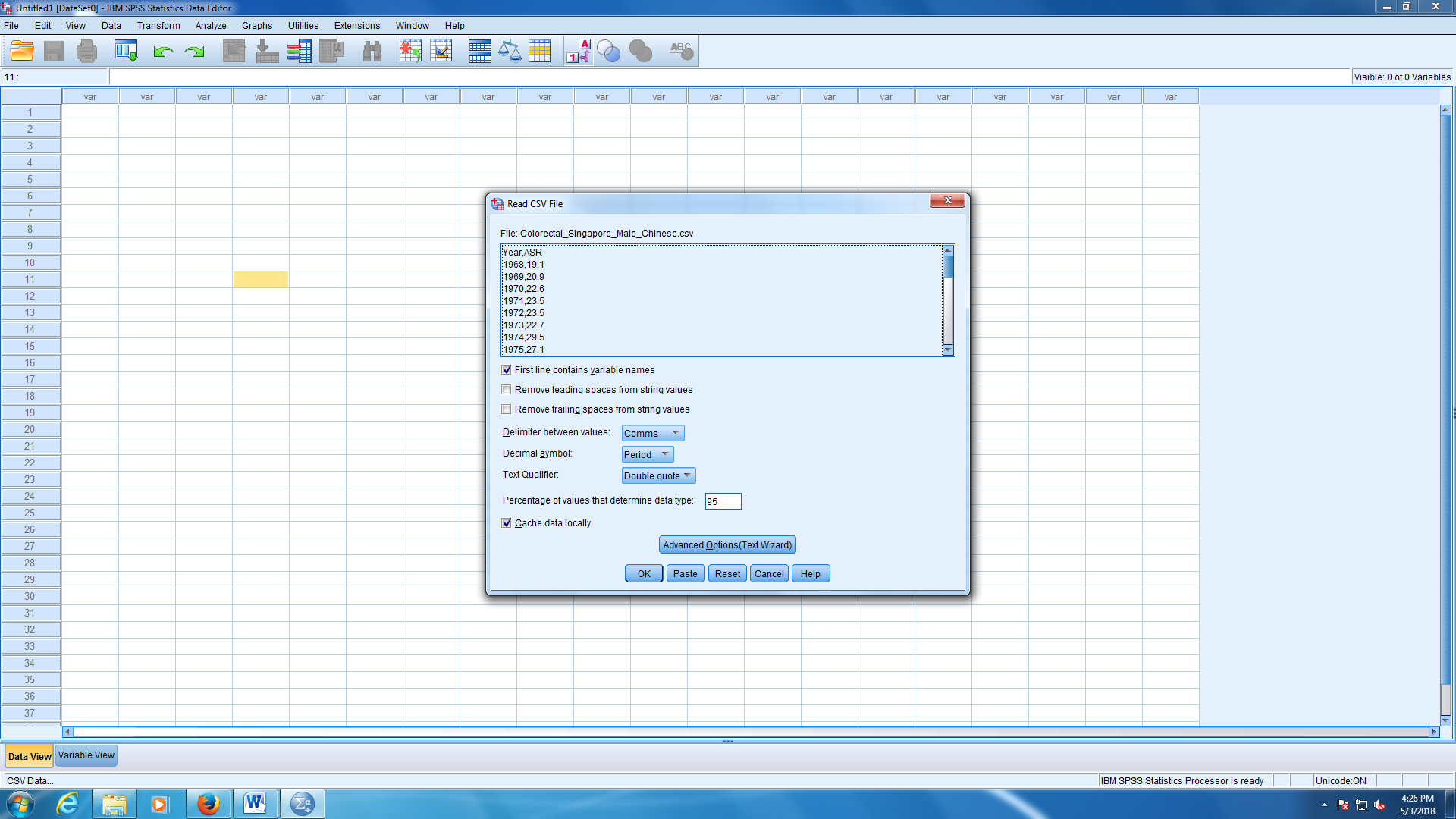


**Step 2:** Recode the outcome, ASR, in the reverse order, and a status variable, status, to indicate no censoring.

- Go to ***Transform > Rank Cases***..
- Under ***the first box***, **select *ASR.***
- Under ***Assign Rank 1 to***, **select** ***Largest Value***.
- **Click** the ***button,
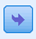
,*** between the box where the variables are and the box under ***Variables,*** to move ***ASR*** to ***Variables***
- **Click** ***OK***.


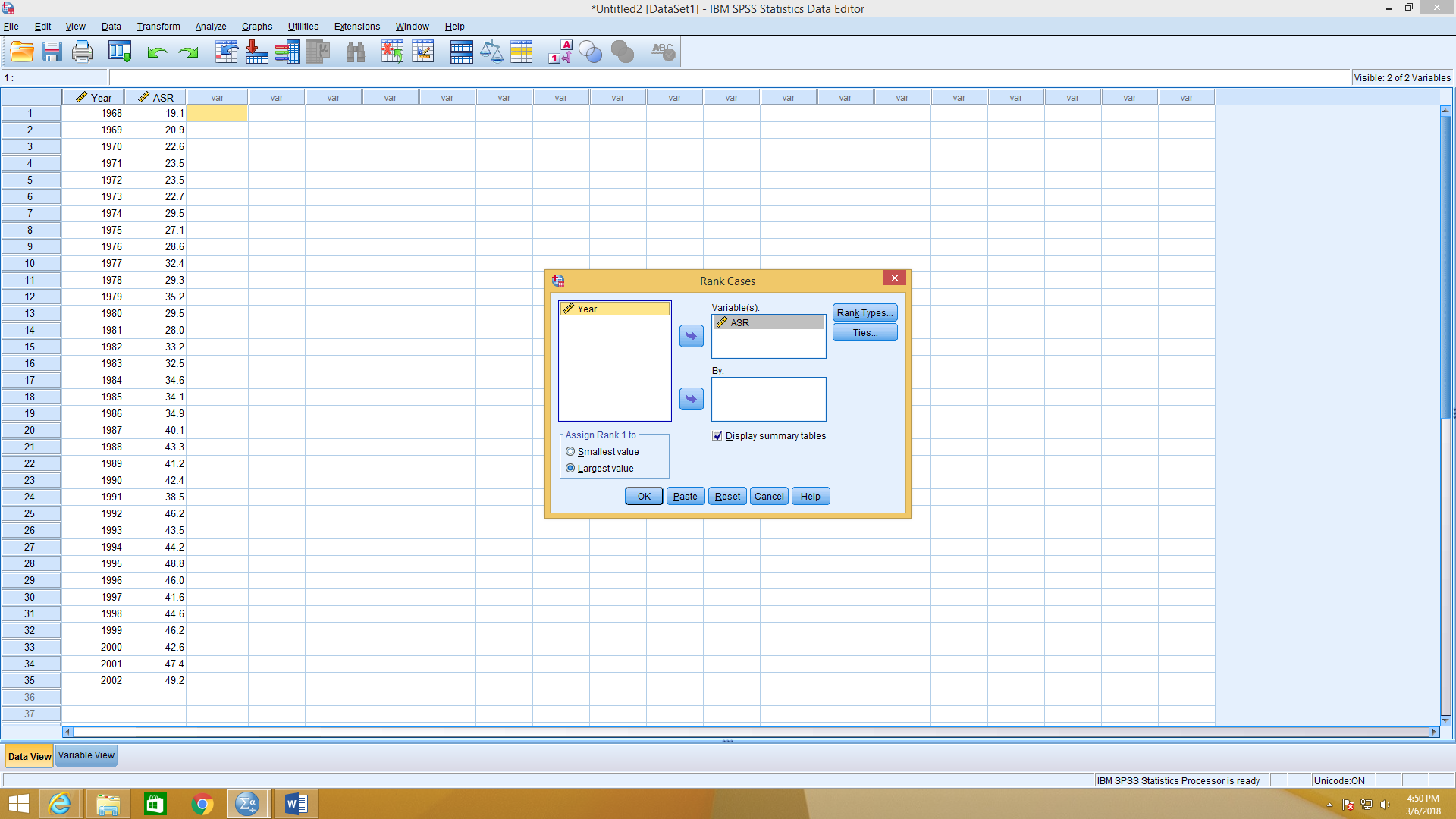


The ranked variable RASR is then created, by default, average ranks will be assigned when there are ties.

| **Created Variables^a^** | | | |
| --- | --- | --- | --- |
| Source Variable | Function | New Variable | Label |
| ASR^b^ | Rank | RASR | Rank of ASR |
| a. Mean rank of tied values is used for ties. | | | |
| b. Ranks are in descending order. | | | |

To create a new variable named status to indicate no censoring for all observations and assign all the values of status to 1.

- Go to ***Transform >Compute Variable***….
- Under ***Target Variable***, **type *Status.***
- Under ***Numeric Expression***, **type *1***.
- **Click** *OK.*


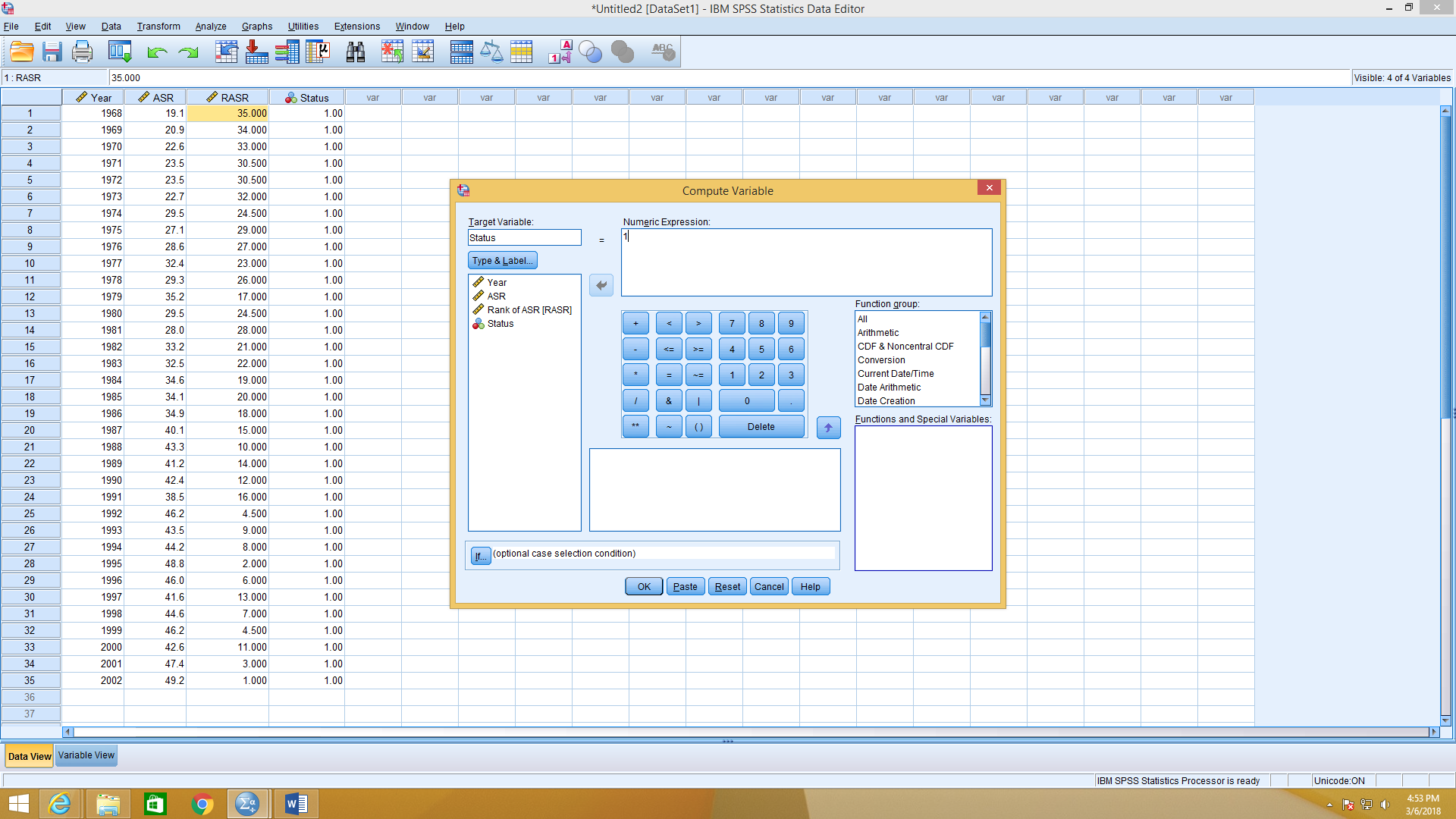


**Step 3:** Implement the RO-logit model by fitting a Cox-PH model to the new outcome, y.

- Go to ***Analyze > Survival > Cox Regression…***
- Under ***Time,* select *y;*** under ***Status,* select *Status;*** and under ***Covariates,* select *Year.***
- **Click *Define Event...*** button.
- Next to **Single value**, **type *1***.
- **Click** ***Continue.***
- **Click *Options*,** then under ***Model diagnostics*,** check ***CI for exp(B) 95%*** to display the 95% CI for the hazard ratios***.***
- **Click** ***Continue*** and then ***OK***.


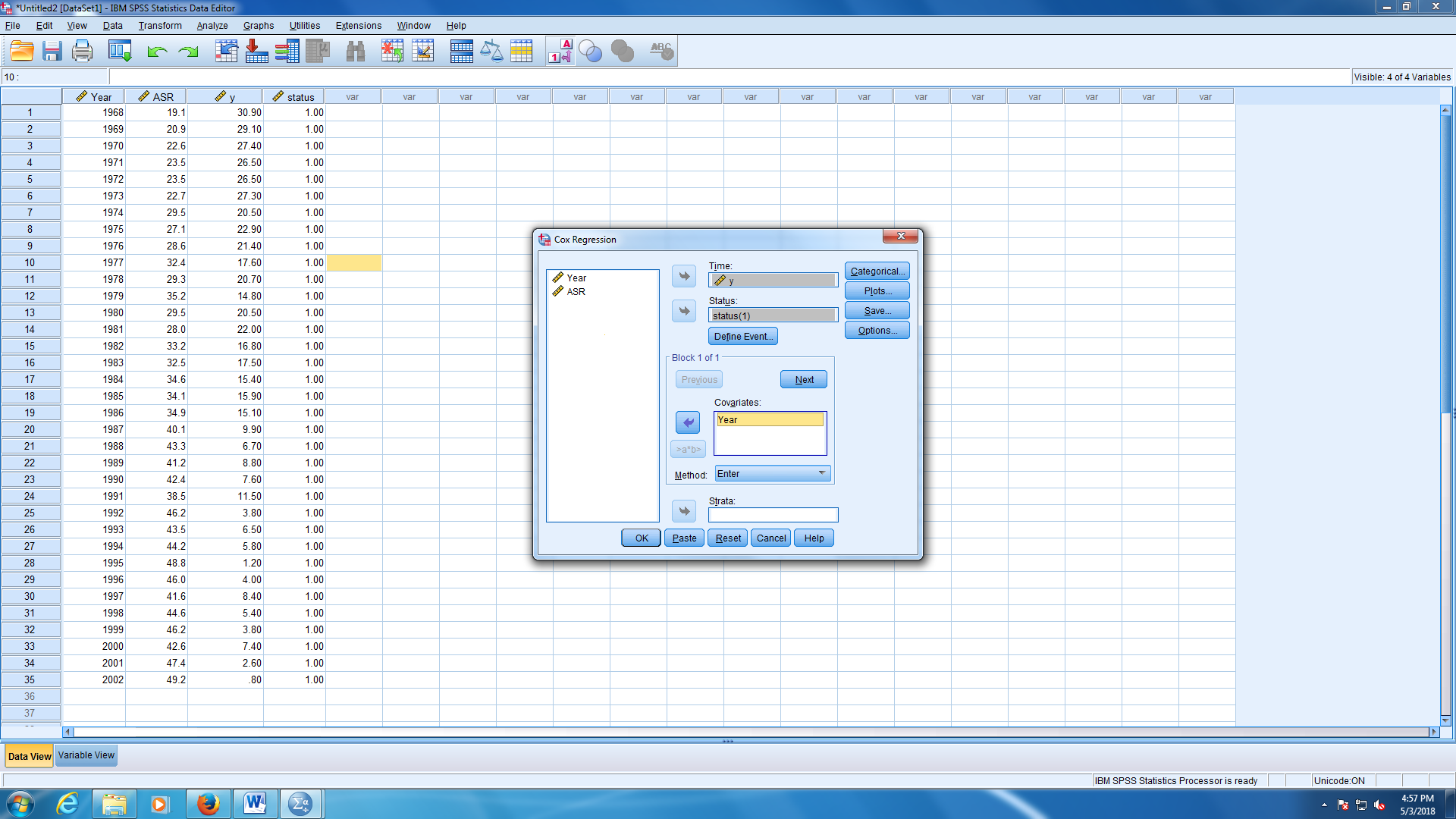

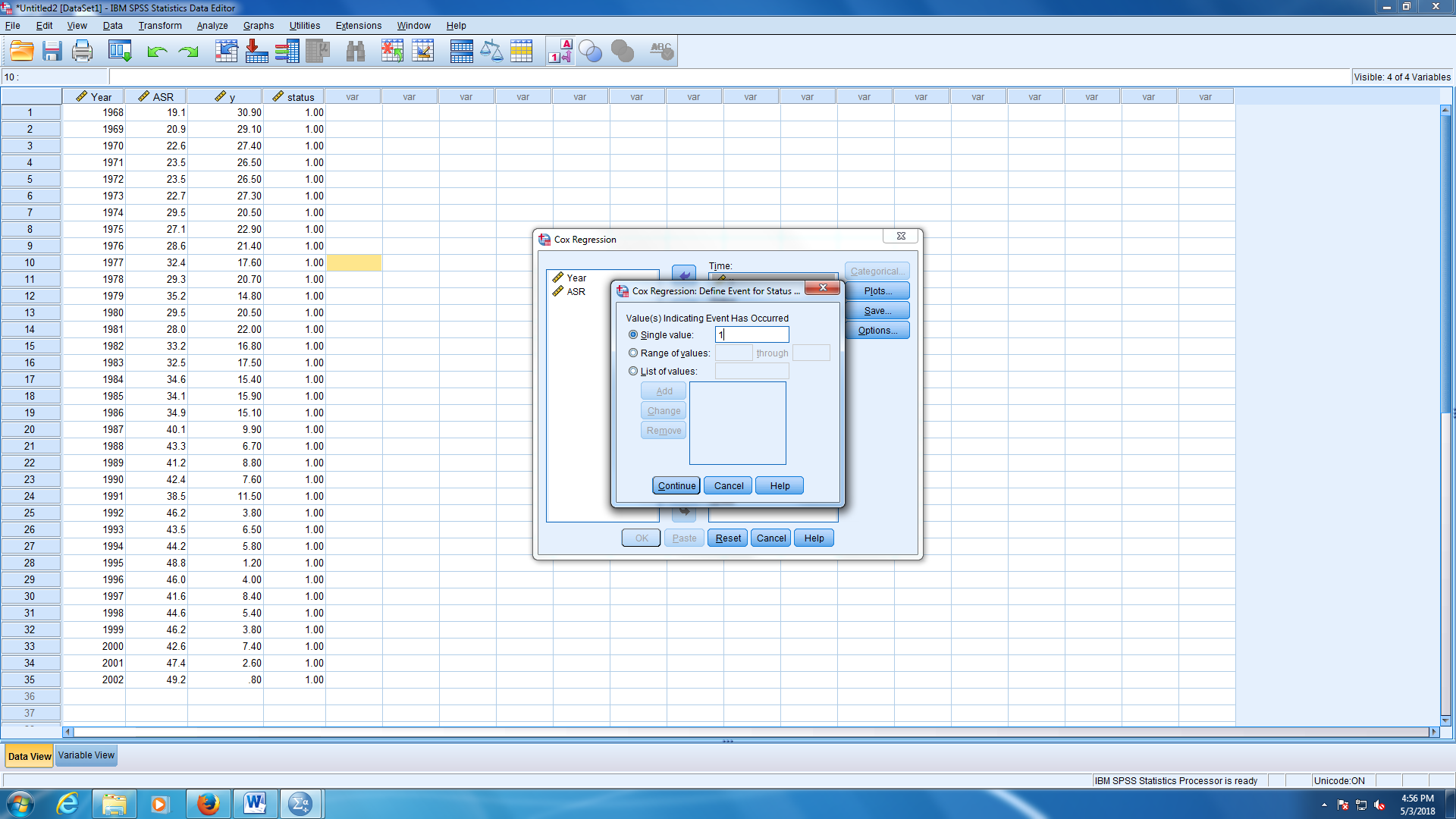


The output from SPSS is as follows

| **Variables in the Equation** | | | | | | | | |
| --- | --- | --- | --- | --- | --- | --- | --- | --- |
|  | B | SE | Wald | df | Sig. | Exp(B) | 95.0% CI for Exp(B) | |
|  |  |  |  |  |  |  | Lower | Upper |
| Year | .296 | .049 | 35.912 | 1 | .000 | 1.345 | 1.221 | 1.482 |

## The estimated coefficient for the RO-logit model is under the “B” entry where the estimate 0.296 (95%CI: 0.200, 0.393; p-value<0.001) suggests a significant positive trend in ASR with the lower and upper 95%CI corresponding to ln(1.221) and ln(1.482) respectively. The SPSS results deviates slightly from those in R, Stata and SAS because the Breslow method is used in SPSS and not Efron method.

# ***Implementation in SPSS via command line***

**Step 1:** Import data

GET DATA /TYPE=TXT

/FILE="path\to\ Colorectal_Singapore_Male_Chinese.csv"

/ENCODING='UTF8'

/DELIMITERS=","

/QUALIFIER='"'

/ARRANGEMENT=DELIMITED

/FIRSTCASE=2

/DATATYPEMIN PERCENTAGE=95.0

/VARIABLES=

Year AUTO

ASR AUTO

/MAP.

CACHE.

EXECUTE.

**Step 2:** Recode the outcome, ASR, in the reverse order, and a status variable, status, to indicate no censoring.

| RANK VARIABLES=ASR (D)  /RANK  /PRINT=YES  /TIES=MEAN.  EXECUTE.  COMPUTE Status=1.  EXECUTE. |
| --- |

**Step 3:** Implement the RO-logit model by fitting a Cox-PH model to the new outcome, y.

| COXREG RASR  /STATUS=Status(1)  /METHOD=ENTER Year  /PRINT=CI(95)  /CRITERIA=PIN(.05) POUT(.10) ITERATE(20).  EXECUTE. |
| --- |

# ***Implementation in SAS***

**Step 1:** Import data.

proc import

datafile="path\to \Colorectal_Singapore_Male_Chinese.csv"

out=dat dbms=csv replace;

getnames=yes;

run;

**Step 2**: Recode the outcome, ASR, in the reverse order.

Proc rank data=dat out=dat_new descending ties=mean;

Var ASR;

Ranks y;

Run;

**Step 3:** Implement the RO-logit model by fitting a Cox-PH model to the new outcome, y.

proc phreg data=dat_new;

model y=year / ties=efron;

run;

The output from SAS for Cox-PH model is as follows:

| **Analysis of Maximum Likelihood Estimates** | | | | | | |
| --- | --- | --- | --- | --- | --- | --- |
| **Parameter** | **DF** | **Parameter Estimate** | **Standard Error** | **Chi-Square** | **Pr > ChiSq** | **Hazard Ratio** |
| **Year** | 1 | 0.29707 | 0.04943 | 36.1260 | <.0001 | 1.346 |

The estimated coefficient for the RO-logit model is under the “Parameter Estimate” entry, which takes value 0.297 (95%CI: 0.201, 0.393; p-value<0.001) which suggests a significant positive trend in ASR where the lower and upper 95%CI is 0.297-1.96×0.049 and 0.297+1.96×0.049 respectively.
